# Telogen Effluvium Following the Treatment of Euglycemic Diabetic Ketoacidosis in a Patient With Heavy Soft Drink Intake: A Case Report

**DOI:** 10.7759/cureus.71863

**Published:** 2024-10-19

**Authors:** Yuta Yoshino, Maho Hayashi

**Affiliations:** 1 Internal Medicine, Saitama Citizens Medical Center, Saitama, JPN

**Keywords:** euglycemic diabetoketoacidosis, luseogliflozin, non-scarring alopecia, sodium-glucose co-transporter 2 inhibitors (sglt2-i), telogen effluvium

## Abstract

Telogen effluvium can be triggered by physical exhaustion, drug use, and emotional stress. However, in approximately 33% of cases, the triggers are unknown. Telogen effluvium can develop two to three months after exposure to triggers and improve three to six months after trigger removal. This case report discusses a man with type 2 diabetes mellitus associated with heavy soft drink intake who developed telogen effluvium following treatment for euglycemic diabetic ketoacidosis with luseogliflozin. A 28-year-old man was hospitalized for euglycemic diabetic ketoacidosis. Two months after discharge, he experienced hair loss that persisted for three months. Physical wasting due to hyperglycemia and ketoacidosis was assumed to have triggered hair loss, resulting in rapid weight loss. Investigating the triggers associated with alopecia is crucial for diagnosing and treating telogen effluvium.

## Introduction

The hair cycle, which consists of growing, involuting, and resting phases [1], causes the hair follicle to renew, grow, and shed repeatedly. Telogen effluvium is a non-inflammatory, non-scarring form of alopecia that results in morbid hair loss due to the hair cycle [2]. Telogen effluvium can be caused by physical exhaustion, drug use, or psychological stress, and alopecia generally develops two to three months after exposure to these triggers [3]. Once alopecia occurs, hair loss improves within three to six months after trigger removal [4]. Investigating the timing of hair loss onset allows for alopecia triggers to be identified and modified [5]. This article reports the case of a man with type 2 diabetes mellitus caused by heavy soft drink intake who developed telogen effluvium following treatment for euglycemic diabetic ketoacidosis (EDKA) with luseogliflozin, a sodium-glucose co-transporter-2 (SGLT2) inhibitor.

## Case presentation

A 28-year-old man, who had undergone a cholecystectomy three years prior, presented to our hospital with dyspnea. The patient was a night-shift part-time worker who had drunk two liters of sweetened soft drinks during the night shift alone. Furthermore, he would have a large serving of soy noodles at almost every meal. He had lost 18 kg over the past three months and experienced thirst. One week before he visited our hospital, a local internist diagnosed him with type 2 diabetes and prescribed luseogliflozin, an SGLT2 inhibitor. His breath had a marked acetone-like odor, and he was tachypneic at the initial examination. Therefore, a saline solution was administered immediately. Laboratory testing revealed normal creatinine, a blood glucose of 174 mg/dL, and an elevated glycated hemoglobin A1c of 16.1%. Blood gas analysis revealed high anion gap metabolic acidosis (pH 7.13, HCO3- 3.6 mmol/L, Na^+^ 129 mmol/L, and Cl^-^ 102 mmol/L). A urinalysis revealed elevated ketones (3+), leading to the diagnosis of EDKA (Table 1).

**Table 1:** Biochemical, urine test, and blood gas analysis. TSH: thyroid-stimulating hormone; GAD: glutamic acid decarboxylase.

Variable	Result	Normal values
Aspartate aminotransferase (U/L)	15	10-40
Alanine aminotransferase (U/L)	12	5-45
Urea nitrogen (mg/dL)	10.6	8-20
Creatinine (mg/dL)	0.65	0.65-1.09
TSH (µIU/mL)	108	0.35-4.94
Free T4 (ng/dL)	0.89	0.70-1.48
Anti-GAD antibody (U/mL)	<5.0	0.0-4.9
Glucose (mg/dL)	174	70-109
Glycated hemoglobin A1c (%)	16.0	4.6-6.2
pH	7.13	7.35-7.45
Na^+^ (mmol/L)	129.0	135-148
Cl^-^ (mmol/L)	102	98-106
HCO3^-^ (mmol/L)	3.6	20-26
Urinary protein	2+	Negative
Urinary glucose	4+	Negative
Urinary ketone	3+	Negative

**Figure 1:**
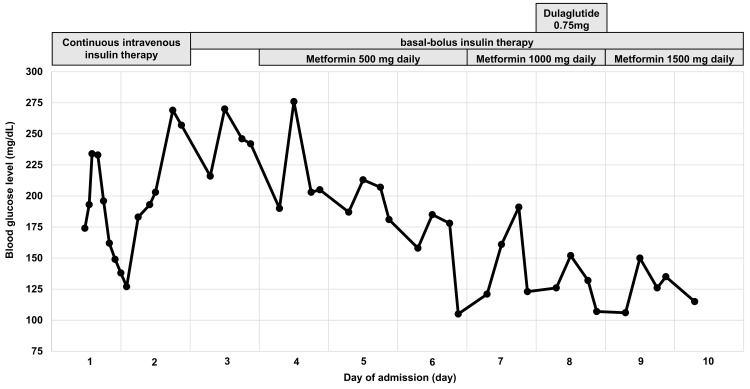
Timeline illustrating blood glucose levels and diabetes treatment during hospitalization.

Continuous intravenous insulin therapy was immediately initiated, and the anion gap normalized on day three of admission. The patient resumed oral intake and was switched to basal-bolus insulin therapy. Metformin 500 mg daily was administered beginning day four, and dulaglutide was injected on day eight of admission, respectively, to avoid EDKA associated with SLGT2 inhibitors. After the metformin dose was increased to 1500 mg daily, the mean premeal blood glucose level stabilized to 130 mg/dL. The patient was instructed to self-inject insulin and was discharged on day 10 of hospitalization (Figure 1).

Two weeks after discharge, basal-bolus insulin therapy was terminated, and glimepiride (0.5 mg) was added daily. The patient complained of hair loss two months after discharge. He noted copious hairs on his pillow on awakening and increased hair loss during hair washing. The patient reported that the hair loss had decreased after three months.

## Discussion

Alopecia can be classified by hair follicle scarring. Cicatricial alopecia is an irreversible form of alopecia caused by the destruction of hair follicles, resulting in damage to follicle stem cells [6]. Non-scarring alopecia, such as alopecia areata [7] and androgenetic alopecia [8], is reversible and caused by hair follicle inflammation or hair cycle abnormalities. Alopecia can also be categorized as a disorder involving the hair cycle, with anagen and telogen effluvium. Telogen effluvium is clinically characterized by hair loss occurring two to three months after a trigger, which can include pregnancy, physical exhaustion, drug use, psychological stress, surgery, and rapid weight loss [3]. The patient in the present case developed type 2 diabetes mellitus due to heavy consumption of soft drinks, resulting in a rapid weight loss of 6 kg per month. Treatment with SGLT2 inhibitors can lead to EDKA and severe wasting, thus triggering telogen effluvium. The treatment of telogen effluvium is dependent on eliminating the trigger. Therefore, the medical history related to the trigger-until approximately three months before the alopecia-must be extracted. In the present case, the trigger associated with hair loss was clear, and the time course was characteristic presentation, allowing for the diagnosis of telogen effluvium.

Telogen effluvium is one of the most common causes of diffuse non-scarring alopecia. However, physicians often use telogen effluvium as a wastebasket diagnosis [2]. Diagnosing telogen effluvium requires laboratory tests for T3, T4, thyroid-stimulating hormone, ferritin, and zinc to exclude endocrine and nutritional disorders [9]. Given that hair loss has also been reported in autoimmune diseases, antinuclear antibody titers should be assessed when other features of autoimmune diseases exist [3]. Acute telogen effluvium is defined as hair loss lasting no longer than six months, and in approximately 33% of cases, the cause remains unknown [10]. The most definitive test to diagnose telogen effluvium is a scalp biopsy, which is usually not required. A scalp biopsy is recommended when hair loss persists for over six months, and multiple biopsies improve diagnostic accuracy [11]. In this case, neither antinuclear antibody analysis nor scalp biopsy was performed because no clinical features suggested autoimmune disease, and the duration of hair loss was approximately three months. Understanding the characteristic clinical course and triggers of acute telogen effluvium can help patients cope with frustration. Chronic telogen effluvium, which develops in association with psychological stress, does not require specific treatment, making the management of alopecia difficult. Explaining and educating patients about the natural history of alopecia is vital for disease management. This includes counseling them regarding their psychological stress [12].

## Conclusions

Telogen effluvium is one of the most common causes of diffuse non-scarring alopecia. Careful extraction of the medical history is required since the triggering event may have occurred approximately three months before the onset of hair loss. In the present case, physical wasting due to hyperglycemia and ketoacidosis was assumed to have triggered hair loss, which was complicated by rapid weight loss. Given the chronology of the hair loss and the characteristic triggers, a clinical diagnosis of telogen effluvium was made without scalp biopsy. Hair is a cosmetically important structure closely associated with personal identity. Psychological stress caused by hair loss may reduce the quality of life, and counseling patients for psychological stress is crucial in cases of long-lasting alopecia.
